# Assessment of industrial cheese ripening using near infrared spectroscopy technique: A scoping review protocol

**DOI:** 10.1371/journal.pone.0335523

**Published:** 2025-11-06

**Authors:** Ana Michell García Varela, Woska Pires da Costa, Danyla Rafaela Oliveira Batista, Vania Silva Carvalho, Wendy Paola Piura, Luis Carlos Cunha Júnior

**Affiliations:** 1 Nutrition Faculty (FANUT), Universidade Federal de Goiás (UFG), Goiânia, GO, Brazil; 2 Research Department, Instituto Federal Goiano - Campus Morrinhos, Morrinhos, GO, Brazil; 3 Medical School, Universidade de São Paulo (USP), São Paulo, SP, Brazil; 4 Instituto de Investigaciones One Health, Universidad Tecnológica Centroamericana (UNITEC), Tegucigalpa, Francisco Morazán, Honduras; Universidad Autonoma de Chihuahua, MEXICO

## Abstract

**Introduction:**

Cheese maturation is an essential stage in dairy production, significantly influencing the sensory quality and market value of the final product. Traditional monitoring methods are often subjective, costly, and rely on destructive sampling, limiting their effectiveness. Near-infrared (NIR) spectroscopy has emerged as a promising, non-destructive, rapid technique capable of providing objective, quantitative data and integration into real-time industrial processes. However, literature still lacks standardized approaches, validation across different cheese types, and comprehensive, methodologically robust reviews to support its broader application. This protocol outlines a scoping review to map and synthesize the available evidence on the use of NIR for monitoring cheese maturation. By investigating its industrial applications, identifying equipment, configurations, and spectral ranges used, exploring data analysis methods, and highlighting reported limitations, challenges, and research gaps.

**Method:**

This scoping review protocol follows the JBI methodology and PRISMA-S guidelines, employing a validated search strategy based on the PRESS 2015 checklist, adapted for use across multiple databases. A comprehensive search will be conducted in Scopus, Web of Science, PubMed, Embase, and FSTA, using a PCC-based strategy. Secondary searches through citation tracking will complement the process. All study designs will be considered without language or date restrictions. Screening and selection will be performed independently by reviewers using the Rayyan software. Data will be extracted and analyzed using descriptive and thematic analysis in NVivo. Methodological quality will be assessed using consolidated checklists. Findings will be presented in narrative, tabular, and diagrammatic formats.

**Conclusion:**

This will be the first systematic synthesis of evidence on the effectiveness of NIR in assessing cheese ripeness, emphasizing its potential for improving production and quality control. By identifying challenges such as the lack of standardization and variability in equipment and models, among others, the review will help define best practices, guide future research, and support the broader adoption of NIR in the dairy industry.

**Trial register:**

OSF Registries, Jul 2, 2025: https://doi.org/10.17605/osf.io/2w4bv.

## 1. Introduction

The cheese industry, valued at billions of dollars annually, is important for global food and nutritional security, sustaining economies and livelihoods in various regions of the world [1]. The success of this sector depends on the ability to produce consistent, high-quality cheeses, which requires precise control of various production processes, especially the ripening phase [2,3]. Cheese ripening is a complex biochemical process involving enzymatic transformations, proteolysis, lipolysis, and the formation of compounds responsible for flavor and aroma development [4–6]. These transformations define the sensory characteristics and quality of the final product. To meet consumer expectations and ensure compliance with quality standards, cheese ripening assessment must be a continuous, systematic process based on objective criteria [7]. Thus, for the product to have adequate and standardized flavor, aroma, and texture, the maturation process requires rigorous management with constant monitoring of the physical-chemical, microbiological, and sensory parameters involved.

Although traditional methods, such as chemical and sensory analyses, are frequently used, they have considerable limitations [8,9]. Chemical analyses are generally time-consuming and laborious, requiring a large number of resources and destructive sampling, which generates waste and prevents continuous monitoring of the same sample throughout the maturation process [10]. Additionally, dependence on the analyst’s experience and sensitivity can lead to inconsistencies and increase the variability of results [11]. Similarly, sensory analysis is susceptible to factors such as individual preferences, evaluator training, and environmental conditions, which make it difficult to obtain consistent, standardized, and objective evaluations [12]. This method is also costly, requires skilled labor and adequate infrastructure, and does not provide a comprehensive characterization of the biochemical and physicochemical parameters necessary to understand the maturation process fully [13].

Near-infrared spectroscopy (NIR) emerges as a promising alternative for assessing the ripeness of industrial cheeses, overcoming several limitations of conventional methods [14]. This technique enables rapid and non-destructive analysis, allowing samples to be evaluated in a few seconds without the need for preparation or destruction [15]. It also allows for continuous monitoring of the same sample throughout the maturation process [16]. In addition, NIR spectroscopy provides objective and quantitative data on the chemical and biochemical composition of cheese, with the potential to increase the accuracy and reliability of evaluations [17,18]. The combination of this technique with statistical methods, such as principal component analysis (PCA), partial least squares (PLS), and linear discriminant analysis (LDA), has proven to be a robust tool for cheese authentication. Its versatility allows it to be applied to different types of cheese and varying stages of maturation, making it an efficient and strategic alternative for the industry [19]. Furthermore, NIR spectroscopy has the potential to be integrated into quality control and production process automation systems, facilitating real-time monitoring and optimization of maturation, which contributes to standardization and product quality assurance [20].

Despite the growing interest in applying NIR spectroscopy to evaluate cheese ripeness, gaps remain in the literature, underscoring the need for further studies [21]. There is no consensus on the most suitable NIR methods and equipment for different types of cheese and stages of ripeness, and further comprehensive research is needed to identify the most effective and efficient approaches [22]. On the other hand, rigorous validation studies are essential to ensure the accuracy and reliability of predictive models under various production conditions. Most studies focus on cheddar-type cheeses [23,24], with limited research exploring the applicability of NIR spectroscopy to a broader range of cheese varieties.

Some studies on the application of NIR spectroscopy for food analysis have already been conducted in countries such as Italy [14], Spain [25], and Ireland [26]. Regarding the specific application of this technique in cheese analysis, studies have been conducted in New Zealand [23], Sweden [24], Italy [21,27], Switzerland [9], and Spain [28]. In addition to these, although there are reviews on the use of NIR spectroscopy in dairy production, it is observed that such reviews address dairy products broadly [16,19,29] or focus specifically on cheese authentication [17,22]. Although the contributions of these reviews are relevant to the dairy industry, gaps remain in the literature, particularly regarding the application of NIR spectroscopy in evaluating cheese maturation. In addition, the reviews we found did not follow a rigorous and transparent methodological design, such as that proposed in a systematic review or scoping protocols, which compromises reproducibility and increases the risk of bias in the selection of included studies.

### 1.1. Study’s objective

Given this context, the main objective of this study protocol is to outline the method for conducting a scoping review aimed at gathering and synthesizing the available evidence on the use of NIR spectroscopy in monitoring cheese maturation, seeking to answer the following questions:

(1) How has this technique been applied in monitoring the cheese maturation process in industry?
(2) Which equipment, configurations, and spectral ranges of NIR spectroscopy have been used for this purpose?
(3) What techniques have been applied in the analysis of data obtained through NIR spectroscopy in this context?
(4) What types of cheese and what stages of maturation are most frequently investigated using this technique?
(5) What are the main limitations, challenges, and gaps identified in the literature on the application of NIR spectroscopy in monitoring cheese maturation?

## 2. Methods

The scoping review has become a recognized scientific method that strives to broadly map a given topic from multiple perspectives, particularly in contexts where the quality assessment of the included studies is not the primary focus. In this sense, the aim is to identify key concepts, categorize research results, and identify gaps. Unlike systematic reviews, scoping reviews are advantageous for answering broad and exploratory research questions, as they encompass all sources, regardless of their quality or methodological rigor. In this context, the purpose is to identify key concepts and categorize research findings. Scoping reviews are especially valuable for exploring new areas of investigation, clarifying essential concepts, or identifying research gaps [30].

A scoping review protocol was developed using the methodological framework [30] and consolidated methods for scoping reviews [31]. Thus, this protocol adheres to the recommendations established for this type of study [32–38]. Accordingly, the development of this protocol was guided by best practice recommendations from the JBI Scoping Review Methodology Group [31], and it will be reported following the Preferred Reporting Items for Systematic Reviews and Meta-Analyses for Protocols (PRISMA-P) 2015 [39] (see S1 File).

### 2.1. Trial registration and ethical issues

This protocol has been preregistered on the Open Science Framework^®^ (OSF^®^ Registries, available at: https://doi.org/10.17605/osf.io/2w4bv) [31,40,41] and published in this scientific journal before data extraction and the initiation of the review to promote transparency [42,43] and ensure the rigor of this study. Any changes made to the protocol during the study will be updated in the trial registration and reported in the final manuscript presenting the results of the scoping review.

In addition to this registration, this study protocol is being published in this scientific journal before data extraction and the start of the review, aiming to ensure methodological rigor and promote research transparency [42,43]. Prospective registration plays a crucial role in disclosing the study’s methodological details, enabling peers to verify whether all procedures were conducted and reported as initially planned [40]. Any amendments to the protocol during the study will be updated in the registration record and reported in the final manuscript presenting the review results. Furthermore, this type of study does not require ethical approval, as it involves no direct interaction with human participants and relies solely on secondary data from previously published sources [44].

### 2.2. Information sources

The evidence sources include quantitative, qualitative, and mixed methods designs studies, which will be considered [42]. Primarily, these sources were indexed in scientific databases and will be selected. All types of study designs, including reviews, meta-analyses, and other types of reports, will be included [36,42]. This systematic scoping will identify and gather scientific evidence from multidisciplinary databases – Scopus^™^ and Web of Science^™^ Core Collection – and specific health databases – MEDLINE/PubMed^®^ via the National Library of Medicine^®^ interface, Embase^™^, and Food Science and Technology Abstracts (FSTA^™^) via EBSCOhost. For systematic searches, a pre-established search string will be employed to identify studies aligned with the focus of this review. Furthermore, the research team will adapt the search strategy for each selected database, incorporating all identified keywords and indexing terms [42,43]. These databases were selected based on identifying relevant studies from prior research to meet the objectives of this study.

**Fig 1:**
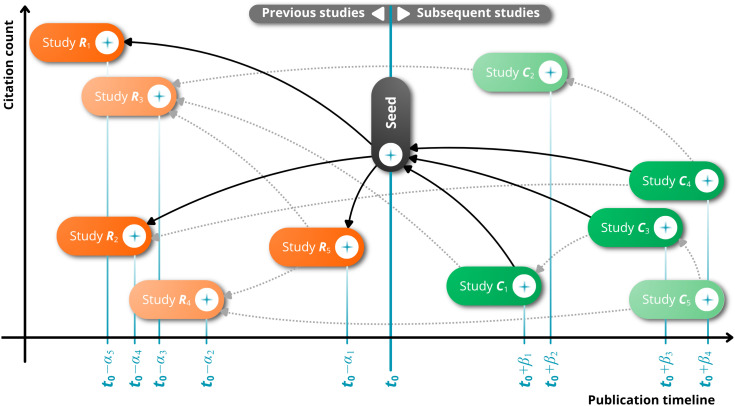
Secondary search process based on ‘seed’ article used to generate the citation map, adapted from Costa et al. (2025) [48]. Note: The black element in the center represents a published study and was selected as the pivot for mapping the evidence; the articles represented by the orange elements – previous studies (R1, R2, and R5) were directly cited by the seed; the articles represented by the green elements are more recent studies (C1, C3, and C4) that cite the seed; and the lighter-colored elements represent studies that resemble the seed, identified as relevant by an artificial intelligence algorithm using the seed as a reference (R3, R4, C2, and C5, linked by dashed lines). The horizontal axis indicates the publication timeline, and the vertical axis refers to the number of citations (higher at the top means a greater number of citations received).

Secondary searches will be conducted through citation tracking to identify additional relevant literature [45] by mapping citations from articles included in the initial phase of the review [46], as well as from review articles that, although excluded, were flagged for this purpose due to their partial relevance to the research topic [47]. This process will be carried out using the Litmaps^®^ platform (https://www.litmaps.com/), which employs artificial intelligence to locate related studies based on a reference article (called seed) and maps scientific evidence through cross-references and citations [47] (Fig 1). For each included article, a search will be performed in Litmaps^®^ using its respective digital object identifier (DOI) [46]. Additionally, all literature will be considered, including specific sources such as Google Scholar^™^, to expand the results as much as possible.

Neither primary nor secondary sources will be restricted by language or publication date [42,43,49]. On the other hand, dissertations, theses, and undergraduate monographs will be excluded. Finally, in each database, to maintain alignment with the search strategy, available filters will be used to exclude studies unrelated to the study’s objective.

### 2.3. Search strategy

The key concepts in the research question and the inclusion criteria established for the study were considered to define the search strategy for this protocol. Some adjustments were made to the syntax of each database to optimize the search and allow for greater precision using advanced search tools. The searches were conducted using the titles, abstracts, and keywords of the scientific databases’ metadata [49].

The mnemonic “PCC” (Population/Participants, Concept, and Context) will be used to predetermine eligibility criteria and guide the identification of relevant studies [31], as it encompasses the most significant elements of the research focus [38]. Considering the specificities of this scoping review, we used “Product” as the P component of this framework. The PCC framework is detailed in Table 1.

**Table 1:** PCC framework.

Component	Definition
P (Product)	Cheeses that undergo a ripening process.
C (Concept)	Use of NIR spectroscopy as an analytical technique.
C (Context)	Industrial-scale production.

**Note:** PCC is a strategy to aid in scoping the review, which defines the key elements of the research, i.e., ‘P’ to delineate the Product, ‘C’ to specify the Concept, and another ‘C’ to detail the Context.

To construct the search string, synonymous and similar terms were considered, as well as British and American English variations, singular and plural forms, and other term-specific nuances. [49]. The search will be performed using the terms specified in the strings (Table 2).

**Table 2:** Strings defined for the primary scientific databases search.

Blocks (PCC)	Keywords used
**#1** P	cheese OR dairy OR mozzarella OR rennet OR camembert OR cheddar OR brie OR stilton OR gouda OR parmesan OR parmigiano OR “parmigiano-reggiano” OR emmental OR emmentaler OR gruyere OR comté OR manchego OR pecorino OR tilsit OR tilsiter OR roquefort OR edam OR colby OR “monterey jack” OR provolone OR asiago OR romano OR jarlsberg OR caciocavallo OR appenzeller OR reblochon OR taleggio OR “murcia al vino” OR gorgonzola OR limburger OR livarot OR feta OR gloucester OR neufchatel
**#2** P	ripened OR ripening OR aged OR aging OR ageing OR affinage OR “flavor development” OR “flavour development” OR “texture development” OR mature OR maturity OR maturation OR matured OR maturing OR “surface-ripened” OR proteolysis OR lipolysis OR “enzymatic activity” OR “chemical composition” OR “volatile compounds” OR “biochemical markers” OR “casein breakdown”
**#3** C	spectroscopy OR “near infrared analysis” OR nir OR “nir-based” OR “infrared reflectance” OR “fourier transform infrared” OR ftir OR “fourier transform near-infrared” OR “ft-nir” OR “visible and near infrared” OR vnir OR “mid-infrared” OR mir OR nirs OR “short-wave near-infrared” OR “short-wave nir” OR “sw-nir” OR swnir OR “multivariate analysis”
**#4** C	industry OR industrial OR “industrial-scale” OR “scale production” OR “production in scale” OR “production-scale” OR “commercial production” OR “commercial-scale” OR “plant-scale production” OR “cheese plant” OR “cheese factory” OR manufacturing OR “quality control” OR “dairy plant” OR “dairy processing facility” OR “milk processing plant” OR “food technology” OR “automated analysis” OR “real-time monitoring” OR “process analytical technology”
**Search string:**	**(#1) AND (#2) AND (#3) AND (#4)**

**Note:** PCC is a strategy to aid in scoping the review, which defines the key elements of the research, i.e., ‘P’ to delineate the Product (the first two blocks), ‘C’ to specify the Concept, and another ‘C’ to detail the Context.

The search strategy was validated using the evidence-based checklist from the Peer Review of Electronic Search Strategies (PRESS 2015) guideline [50]. The syntax was subsequently adapted to meet the specific requirements of each database, thereby broadening the scope and capturing relevant studies. Based on the systematic search string, data extraction will be conducted on a unique day using metadata files generated directly from the database platforms. The entire extraction process will be documented following the PRISMA for Search (PRISMA-S) guidelines [51] (see S2 File). Finally, exhaustive searches in secondary data sources may occur at various moments; as these are not part of a systematic process, detailed documentation may not be feasible.

### 2.4. Eligibility criteria

This systematic review will include relevant articles without language restrictions or publication dates that meet the inclusion criteria. Some eligible articles may be excluded if they meet at least one of the predefined exclusion criteria [49,52], as detailed below.

Inclusion criteria:

(i1) Evidence from studies retrieved from scientific databases using the strategy previously defined with a primary systematic search string or by secondary searches.
(i2) Peer-reviewed journal articles published as original studies [52,53], and technical reports.
(i3) Studies on the evaluation of cheese ripening on an industrial scale using NIR spectroscopy.

Exclusion criteria:

(e1) Duplicates: If multiple articles have been published by the same author, on the same dataset, and on the same topic, only the most comprehensive among them will be considered. Duplicates will be removed following Bramer’s method [54], and we will conduct a manual review to confirm their exclusion [52,55].
(e2) Studies focused on artisanal, homemade, or non-industrial cheeses, or studies without specification of cheese type.
(e3) Studies that used analytical methods involving NIR spectroscopy without addressing the cheese ripening process.
(e4) Dissertations, theses, and reviews.
(e5) Studies that are not fully available in the searched databases and cannot be accessed even after attempts to contact the authors have been made [36,52,53].
(e6) Articles written in a restricted language that cannot be appropriately translated [56]. This criterion will apply only after exhausting all translation options – including international collaborators, artificial intelligence tools, and specialized services – and will be reported in the review findings [49].
(e7) Studies with retraction records [56].

Regarding the last criterion described, considering the substantial variability in how retractions are disseminated – which may result in retracted articles still being cited and included in studies – it is necessary to perform retraction checks [57]. The validity of eligible studies will be assessed, and any retraction records will be identified via the Scite tool – an acronym for “*Smart citation index*” (available at https://scite.ai/) [58]. This tool offers several functionalities, including the ability to determine whether a given article has been retracted or has received critical citations [59].

### 2.5. Screening processed

**Fig 2:**
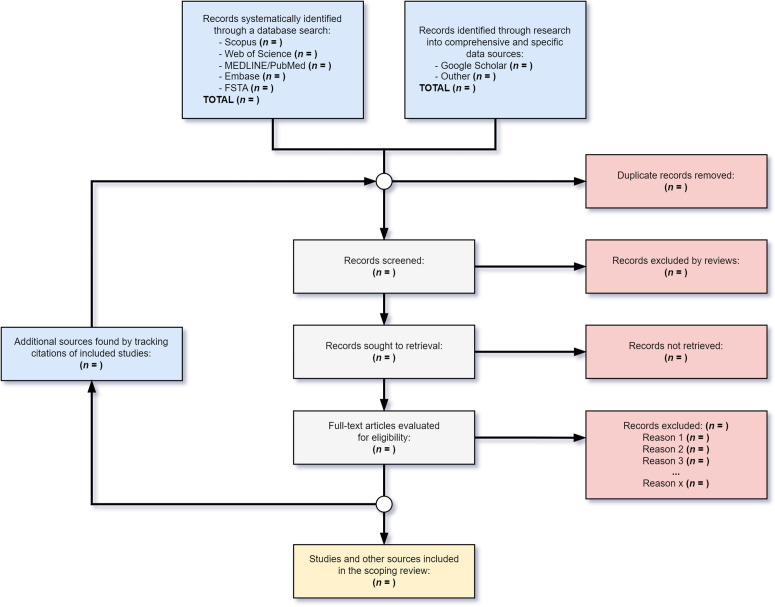
Scoping review flow diagram, adapted by Chaves et al. (2025) [48].

The screening process outlined in this protocol presents a priori description of the planned methodological steps for conducting the scoping review. All stages of the review process are illustrated in the flow diagram proposed for this type of study (Fig 2), which will guide reviewers from the selection of information sources through all intermediate stages – including the recursive citation tracking step mentioned earlier – up to the inclusion of studies and relevant information in the scoping review. The research questions will guide decisions and, most importantly, the eligibility criteria [48]. Further, all findings from this process will be documented for inclusion in the final manuscript presenting the scoping review results.

Before the screening process, once the metadata from the scientific databases have been imported into the Rayyan^®^ software (Rayyan Systems Inc., Cambridge, MA, USA), the primary reviewer will execute the deduplication process [46]. This online tool (https://www.rayyan.ai/) was designed to facilitate the conduct of systematic reviews [60], scoping reviews, and other types of reviews. Thus, this software will perform all stages of the scoping review, with the reviewers’ blinding feature activated for evaluation [56]. It includes support for auto-resolving duplicated studies, determining the inclusion or exclusion of studies based on eligibility criteria, and labeling to provide more detailed information about their decisions through facilities [61].

During the first stage of screening studies extracted from primary sources, each study will be screened by reading the title and abstract to ensure alignment with the inclusion criteria, and each hit will then be reviewed independently by two reviewers [60,62]. The study co-authors will act as reviewers, and if necessary, new reviewers may be added to ensure that the study remains manageable and that the scoping review remains feasible [62]. A third independent senior reviewer will resolve any potential discrepancies [41,49]. In terms of secondary sources, the entire process will be conducted by the first author, who will serve as the primary reviewer for this scoping review. In cases of uncertainty, a senior reviewer may be consulted to support decision-making.

In the second stage, the reviewers will read articles marked for full-text review to ensure that they meet the inclusion criteria and do not fall under any exclusion criteria [60,63]. Any discrepancies will be reviewed by an additional team member, who will decide on the article’s inclusion [63]. If a study is unavailable, one reviewer will contact the corresponding author to request the full text [41] and, if necessary, the related dataset.

Finally, all decisions made for each study will be duly labeled to ensure precise, transparent, and complete documentation of the entire process, including both inclusion and exclusion criteria, at both the first and second stages of the review.

### 2.6. Review training

A pilot assessment will be conducted to train the reviewers involved in this study under the supervision of an experienced researcher. This training session objectives to standardize the screening decisions based on the pre-established eligibility criteria for this review [47]. It will also include guidance on using the Rayyan^®^ software, ensuring reviewers become familiar with its features and functionalities, as well as promote a standardized analysis process and help reviewers correctly label the studies at each review stage [48].

### 2.7. Data extraction

All data extracted from the evidence sources will be entered into a summarized table that includes information such as the study, authors, publication year, conflicts of interest, source, objective, cheese type, methodology, ripening method, NIR spectroscopy technique used, ripening parameters evaluated (e.g., proteolysis and lipolysis), and other relevant findings (see S3 Table). This table will be reviewed and adjusted as necessary during the data extraction process [36,60,64]. If a study is unavailable, one reviewer will contact the corresponding author to request the full text [41] and, if necessary, the related dataset. The full extracted evidence will be compiled as supplementary material and made available alongside the scoping review publication as “Evaluated Data”.

### 2.8. Data analysis

The data will be inductively coded in NVivo^®^ (QSR International Pty Ltd., Melbourne, VIC, Australia) using various approaches [62,65]. This coding will enable us to capture the diverse methods, applications, and strategies associated with the use of NIR spectroscopy in monitoring cheese maturation. The results will be synthesized narratively, aiming to concisely describe the characteristics, advantages, and limitations of the approaches identified. Key aspects to be examined include the types of cheese evaluated, the stages of maturation addressed, the equipment and spectral ranges used, as well as the statistical and computational methods applied to analyze the data generated through NIR spectroscopy. This approach will enable a critical analysis of the evolution and application of this technology in the industrial cheese sector, highlighting existing methodological gaps and challenges.

Data analysis will consist of basic descriptive [42] and reflexive thematic analysis [66]. The six thematic analysis steps were followed [67]: i) familiarization with the data through repeated reading and annotation; ii) generation of initial codes through intense open-coding of data to generate an initial coding frame based on thematic categories rooted in the data; iii) identification of themes, through a detailed review of the coding frame to sort codes into potential themes; iv) review of themes, through refinement of the developing themes; v) definition and refinement of themes, through exploration of relationships within and between codes, and revision of thematic definitions; and vi) writing of the study findings.

The results will be presented in tabular and diagrammatic formats to align with the review objective. A narrative summary will accompany the tabulated and charted results, describing how these results relate to the review objective and questions.

### 2.9. Methodological quality

Although assessing the risk of bias is not a mandatory component of scoping reviews, it can enhance the value of the evidence by providing insight into the breadth, diversity, and characteristics of the existing literature on a given topic [34,68]. Nevertheless, in this review, the inclusion of studies will be based exclusively on predefined eligibility criteria. Subsequently, the methodological quality of each included study will be evaluated individually. The quality of the studies included will be assessed using two checklists. For qualitative studies, the Critical Appraisal Skills Program (CASP) will be employed for critical appraisal [69]. For quantitative studies, the Downs and Black checklist [70] will be used for assessment. The studies that adopted mixed methods will be evaluated from both perspectives, with the qualitative results being analyzed using CASP and the quantitative results using the Downs and Black checklist [71].

The CASP checklist is widely recognized for evaluating the risk of bias in qualitative research [72,73]. It consists of ten questions organized into three sections [72]: (A) “*Are the results of the study valid?*”, (B) “*What are the results?*”, and (C) “*Will the results be useful locally?*”. Nine questions receive objective scores: “*yes*” (2 points), “*can’t tell*” (1 point), or “*no*” (0 points), resulting in a maximum possible score of 18 [74]. The final question is open-ended and requires a qualitative response. Overall quality was rated using a three-star system [74]: one star (0–6 points), two stars (7–12 points), and three stars (13–18 points).

The Downs and Black checklist comprises 27 items [70]; however, to align with the specific scope of this review, we will apply an abridged 10-item version (items 1–3, 7, 10–12, 16, 18, and 20) [71]. The quality of each study will be calculated as a percentage; scores ≥70% will indicate a low risk of bias, whereas scores <70% will indicate a high risk of bias [70].

### 2.10. Presentation of results

The study screening process will be described using narrative, tabular, and visual formats to demonstrate how the results align with the objectives and research questions of the review [42]. The elements of the PCC framework’s inclusion criteria will be considered to guide the best format for presenting the review results to the audience [34]. Thus, the results will be organized in a spreadsheet and included as supplementary material. If necessary, the findings will be categorized and subcategorized objectively and concisely. Additionally, as appropriate, quantitative data may be transformed into themes or categories [75]. The evidence gathered may also be presented using figures, diagrams, or other visual elements that best illustrate the clustering of factors and emerging patterns and trends [76,77]. Presenting the results in a suitable and detailed format will allow the reviewers to identify gaps in the literature and map the available evidence [34].

### 2.11. Ethics and dissemination

Ethical approval is typically not required for scoping studies, as they often analyze previously published studies or publicly available evidence (i.e., secondary data). The results and conclusions obtained will be published in a peer-reviewed journal to contribute to advancing the research area and supporting further investigation.

## 3. Discussion

This investigation will be the first scoping review to summarize the evidence on the effectiveness of NIR spectroscopy in assessing the ripeness and flavor of industrial cheeses based on studies published in scientific journals. This review will aim to understand the potential of NIR spectroscopy in enhancing cheese production processes and quality control, as well as to identify best practices for its application in the industry [3,4]. In this context, NIR spectroscopy provides a fast and non-destructive method of analyzing cheese maturation [14], enabling continuous monitoring throughout the process [15], and providing real-time data on the biochemical and physicochemical properties of cheeses [16]. As a result, producers and manufacturers will be able to ensure greater consistency in product quality and more accurately meet consumer expectations [8].

The application of a non-destructive analysis technique also allows the same sample to be monitored over time, reducing waste and costs associated with conventional methods, in which the sample is damaged or needs to be discarded after analysis [17]. In addition, advances in the use of NIR spectroscopy depend on defining the most appropriate methods and equipment for different types of cheese and stages of maturation [23], which contributes to the development of standardized protocols applicable to the industry [24]. Standardization will enable NIR assessments to generate consistent and comparable results across different production units [21], thereby facilitating quality monitoring on an industrial scale.

On the other hand, the lack of uniformity in the techniques, statistical models, and NIR equipment used can generate variations in the results, impacting the effectiveness of spectroscopy for different types of cheese and stages of maturation [12]. Currently, there is no consensus on the most appropriate procedures for these applications [19]. Added to this is the challenge of the diversity of existing cheeses, since much of the research focuses on a small number of varieties, especially cheddar cheese [20]. Therefore, there is a need for studies that evaluate the applicability of NIR spectroscopy in a broader range of cheeses, considering their compositional and technological characteristics [11]. Finally, validation studies are needed to consider different processing conditions, environments, and production scales to ensure the accuracy, robustness, and reliability of the developed models [9].

### 3.1. Strengths and limitations

The strengths of this study include a comprehensive evaluation of the application of NIR spectroscopy across a wide range of cheese types and stages of maturation [10], which contributes to the development of standardized NIR methods [7,8], the optimization of production processes, and the enhancement of quality control measures [4]. These include the use of consolidated software to support the entire scoping review process, as well as a comprehensive search strategy that covers multiple scientific databases, including multidisciplinary and broad-scope sources. Additionally, the use of a recursive search strategy through citation tracking of the included studies enhances the retrieval of relevant evidence that may not be captured by the traditional search string alone. By identifying best practices and mapping existing gaps, this review can guide future research and foster the broader and more qualified adoption of NIR spectroscopy in the dairy industry [21]. The assessment of bias risk and evidence quality will enable the identification of studies with more consistent and applicable results, as well as indicate where further methodological robustness is still needed.

Although there are limitations and challenges in applying NIR spectroscopy to evaluate cheese ripeness, the benefits associated with its speed, non-destructive nature, and the possibility of continuous monitoring reinforce its potential as a promising technique for the industry [23]; therefore, this scoping review will gather evidence that can support decision-making in both academia and the productive sector, consolidating itself as a reference for this area of study. This evidence will enable the expanded use of NIR spectroscopy in cheese production, allowing manufacturers to gain greater control over the ripening process and ensure that consumers receive products with quality, flavor, and sensory characteristics that meet expected standards [17].

## Supporting information

S1 File PRISMA-P checklist for the scope review protocol. (PDF)

S2 File Details of Boolean search string used for each database. (PDF)

S3 Table Data extraction summary form of the studies included in this scoping review. (PDF)
