# Supplementary material for: Assessment of industrial cheese ripening using near infrared spectroscopy technique: A scoping review protocol
Source: PLoS One. 2025 Nov 6;20(11):e0335523. doi: 10.1371/journal.pone.0335523 (PMC12591493; doi:10.1371/journal.pone.0335523)
Supplement: S1 File — (PDF) [file pone.0335523.s001.pdf]

**Supplementary Material S1.** PRISMA-P checklist for the scope review protocol

| Section/topic | Item nº | Checklist item | Information reported | | Page |
|---|---|---|---|---|---|
| | | | **Yes** | **No** | |
| **ADMINISTRATIVE INFORMATION** | | | | | |
| Title: | | | | | |
| Identification | 1a | Identify the report as a protocol of a systematic review – in the case of this study, a 'scoping review protocol'. | ✓ | | 1 |
| Update | 1b | If the protocol is for an update of a previous review, identify as such. | | ✓ | N/A |
| Registration | 2 | If registered, provide the name of the registry (such as PROSPERO) and registration number. | | ✓ | N/A |
| Authors: | | | | | |
| Contact | 3a | Provide name, institutional affiliation, e-mail address of all protocol authors; provide physical mailing address of corresponding author. | ✓ | | 1 |
| Contributions | 3b | Describe contributions of protocol authors and identify the guarantor of the review. | ✓ | | 16 and 17 |
| Amendments | 4 | If the protocol represents an amendment of a previously completed or published protocol, identify as such and list changes; otherwise, state plan for documenting important protocol amendments. | | ✓ | N/A |
| Support: | | | | | |
| Sources | 5a | Indicate sources of financial or other support for the review. | | ✓ | No external funding. |
| Sponsor | 5b | Provide name for the review funder and/or sponsor. | | ✓ | No external funding. |
| Role of sponsor or funder | 5c | Describe roles of funder(s), sponsor(s), and/or institution(s), if any, in developing the protocol. | | ✓ | No external funding. |
| **INTRODUCTION** | | | | | |
| Rationale | 6 | Describe the rationale for the review in the context of what is already known. | ✓ | | 2–4 |
| Objectives | 7 | Provide an explicit statement of the question(s) the review will address with reference to participants, interventions, comparators, and outcomes (PICO) – in the case of this study, the questions should be addressed with reference to their key elements (e.g., population or participants, concepts, and context) or other relevant key elements used to conceptualize the review questions and/or objectives, preferably using the PCC framework. | ✓ | | 4 |

| Section/topic | Item nº | Checklist item | Information reported | | Page |
|---|---|---|---|---|---|
| | | | Yes | No | |
| **METHODS** | | | | | |
| Eligibility criteria | 8 | Specify the study characteristics (such as PICO, study design, setting, time frame – in the case of this study, based on the PCC framework) and report characteristics (such as years considered, language, publication status) to be used as criteria for eligibility for the review. | ✓ | | 7–10 |
| Information sources* | 9 | Describe all intended information sources (such as electronic databases, contact with study authors, trial registers or other grey literature sources) with planned dates of coverage. | ✓ | | 5, 6, and 9 |
| Search strategy | 10 | Present draft of search strategy to be used for at least one electronic database, including planned limits, such that it could be repeated. | ✓ | | 7–8, and Supplementary Information S2 |
| Study records: | | | | | |
| Data management | 11a | Describe the mechanism(s) that will be used to manage records and data throughout the review. | ✓ | | 9–12 |
| Selection process | 11b | State the process that will be used for selecting studies (such as two independent reviewers) through each phase of the review (that is, screening, eligibility and inclusion in meta-analysis). | ✓ | | 10 and 11 |
| Data collection process | 11c | Describe planned method of extracting data from reports (such as piloting forms, done independently, in duplicate), any processes for obtaining and confirming data from investigators. | ✓ | | 12, 13, and Supplementary Information S3 |
| Data items | 12 | List and define all variables for which data will be sought (such as PICO items, funding sources – in the case of this study, based on the PCC framework), any pre-planned data assumptions and simplifications. | ✓ | | 12, 13, and Supplementary Information S3 |
| Outcomes and prioritization | 13 | List and define all outcomes for which data will be sought, including prioritization of main and additional outcomes, with rationale. | ✓ | | 12 |
| Risk of bias in individual studies | 14 | Describe anticipated methods for assessing risk of bias of individual studies, including whether this will be done at the outcome or study level, or both; state how this information will be used in data synthesis – in the case of this study, CASP will be utilized to evaluate qualitative studies, and the Downs and Black checklist will be used for quantitative studies. | ✓ | | 14 and 15 |

| Section/topic | Item nº | Checklist item | Information reported | | Page |
|---|---|---|---|---|---|
| | | | Yes | No | |
| Data synthesis | 15a | Describe criteria under which study data will be quantitatively synthesized. | | ✓ | N/A |
| | 15b | If data are appropriate for quantitative synthesis, describe planned summary measures, methods of handling data and methods of combining data from studies, including any planned exploration of consistency (such as I2, Kendall's τ). | | ✓ | N/A |
| | 15c | Describe any proposed additional analyses (such as sensitivity or subgroup analyses, meta-regression). | | ✓ | N/A |
| | 15d | If quantitative synthesis is not appropriate, describe the type of summary planned. | ✓ | | 12, 13, and Supplementary Information S3 |
| Meta-bias(es)** | 16 | Specify any planned assessment of meta-bias(es) (such as publication bias across studies, selective reporting within studies). | | ✓ | N/A |
| Confidence in cumulative evidence** | 17 | Describe how the strength of the body of evidence will be assessed (such as GRADE). | | ✓ | N/A |

**Notes:** PRISMA-P 2015 is a checklist described as the Preferred Reporting Items for Systematic Review and Meta-Analysis Protocols. It is strongly recommended that this checklist be read in conjunction with the PRISMA-P Explanation and Elaboration for important clarification on the items. The copyright for PRISMA-P (including checklist) is held by the PRISMA-P Group and is distributed under a Creative Commons Attribution Licence 4.0. PICO is an abbreviation for Population, Intervention, Comparison, and Outcome; GRADE is an acronym for Grading of Recommendations Assessment, Development, and Evaluation; and CASP refers to the Critical Appraisal Skills Programme. * Sources of evidence will be compiled from bibliographic databases and through recursive citation tracking (i.e., secondary searches) to identify additional relevant literature by mapping citations from articles included in the initial step of the review. ** In scoping reviews, assessing meta-bias(es) and the overall confidence in cumulative evidence is not mandatory, as the objective is not to produce clinical recommendations or causal inferences, but rather to map the scope, variety, and key characteristics of the existing literature. If any information is not reported or not evaluated, use the abbreviation 'N/A' (not applicable) in the column entitled Page.
