# Supplementary material for: Assessment of industrial cheese ripening using near infrared spectroscopy technique: A scoping review protocol
Source: PLoS One. 2025 Nov 6;20(11):e0335523. doi: 10.1371/journal.pone.0335523 (PMC12591493; doi:10.1371/journal.pone.0335523)
Supplement: S2 File — (PDF) [file pone.0335523.s002.pdf]

Supplementary Material S2. Details of Boolean search string used for each database

Scopus™ via native interface	
**Blocks and returns**	**Search strings**
#1 (Return: ‹n_1›)	TITLE-ABS-KEY(cheese OR dairy OR mozzarella OR rennet OR camembert OR cheddar OR brie OR stilton OR gouda OR parmesan OR parmigiano OR "parmigiano-reggiano" OR emmental OR emmentaler OR gruyere OR comté OR manchego OR pecorino OR tilsit OR tilsiter OR roquefort OR edam OR colby OR "monterey jack" OR provolone OR asiago OR romano OR jarlsberg OR caciocavallo OR appenzeller OR reblochon OR taleggio OR "murcia al vino" OR gorgonzola OR limburger OR livarot OR feta OR gloucester OR neufchatel)
#2 (Return: ‹n_2›)	TITLE-ABS-KEY(ripened OR ripening OR aged OR aging OR ageing OR affinage OR "flavor development" OR "flavour development" OR "texture development" OR mature OR maturity OR maturation OR matured OR maturing OR "surface-ripened" OR proteolysis OR lipolysis OR "enzymatic activity" OR "chemical composition" OR "volatile compounds" OR "biochemical markers" OR "casein breakdown")
#3 (Return: ‹n_3›)	TITLE-ABS-KEY(spectroscopy OR "near infrared analysis" OR nir OR "nir-based" OR "infrared reflectance" OR "fourier transform infrared" OR ftir OR "fourier transform near-infrared" OR "ft-nir" OR "visible and near infrared" OR vnir OR "mid-infrared" OR mir OR nirs OR "short-wave near-infrared" OR "short-wave nir" OR "sw-nir" OR swnir OR "multivariate analysis")
#4 (Return: ‹n_4›)	TITLE-ABS-KEY(industry OR industrial OR "industrial-scale" OR "scale production" OR "production in scale" OR "production-scale" OR "commercial production" OR "commercial-scale" OR "plant-scale production" OR "cheese plant" OR "cheese factory" OR manufacturing OR "quality control" OR "dairy plant" OR "dairy processing facility" OR "milk processing plant" OR "food technology" OR "automated analysis" OR "real-time monitoring" OR "process analytical technology")
#5 (Return: ‹n_5›)	(#1) AND (#2) AND (#3) AND (#4)
	**Search refinement**: ⟨⟨Not applied⟩⟩ **Return after refinement**: ‹*N*› studies in the test carried out in ‹*date*›.

Web of Science™ Core Collection via native interface	
**Blocks and returns**	**Search strings**
#1<br>(Return: ‹n_1›)	TS=(cheese OR dairy OR mozzarella OR rennet OR camembert OR cheddar OR brie OR stilton OR gouda OR parmesan OR parmigiano OR "parmigiano-reggiano" OR emmental OR emmentaler OR gruyere OR comté OR manchego OR pecorino OR tilsit OR tilsiter OR roquefort OR edam OR colby OR "monterey jack" OR provolone OR asiago OR romano OR jarlsberg OR caciocavallo OR appenzeller OR reblochon OR taleggio OR "murcia al vino" OR gorgonzola OR limburger OR livarot OR feta OR gloucester OR neufchatel)
#2<br>(Return: ‹n_2›)	TS=(ripened OR ripening OR aged OR aging OR ageing OR affinage OR "flavor development" OR "flavour development" OR "texture development" OR mature OR maturity OR maturation OR matured OR maturing OR "surface-ripened" OR proteolysis OR lipolysis OR "enzymatic activity" OR "chemical composition" OR "volatile compounds" OR "biochemical markers" OR "casein breakdown")
#3<br>(Return: ‹n_3›)	TS=(spectroscopy OR "near infrared analysis" OR nir OR "nir-based" OR "infrared reflectance" OR "fourier transform infrared" OR ftir OR "fourier transform near-infrared" OR "ft-nir" OR "visible and near infrared" OR vnir OR "mid-infrared" OR mir OR nirs OR "short-wave near-infrared" OR "short-wave nir" OR "sw-nir" OR swnir OR "multivariate analysis")
#4<br>(Return: ‹n_4›)	TS=(industry OR industrial OR "industrial-scale" OR "scale production" OR "production in scale" OR "production-scale" OR "commercial production" OR "commercial-scale" OR "plant-scale production" OR "cheese plant" OR "cheese factory" OR manufacturing OR "quality control" OR "dairy plant" OR "dairy processing facility" OR "milk processing plant" OR "food technology" OR "automated analysis" OR "real-time monitoring" OR "process analytical technology")
#5<br>(Return: ‹n_5›)	(#1) AND (#2) AND (#3) AND (#4)
	**Search refinement**:<br>⟨⟨Not applied⟩⟩<br>**Return after refinement**:<br>‹*N*› studies in the test carried out in ‹*date*›.

MEDLINE/PubMed® via interface of the National Library of Medicine® (NLM®)

Blocks and returns	Search strings
#1 (Return: ‹n_1›)	(cheese[MeSH Terms] OR cheese[tiab] OR dairy[tiab] OR mozzarella[tiab] OR rennet[tiab] OR camembert[tiab] OR cheddar[tiab] OR brie[tiab] OR stilton[tiab] OR gouda[tiab] OR parmesan[tiab] OR parmigiano[tiab] OR "parmigiano-reggiano"[tiab] OR emmental[tiab] OR emmentaler[tiab] OR gruyère[tiab] OR gruyere[tiab] OR comté[tiab] OR manchego[tiab] OR pecorino[tiab] OR tilsit[tiab] OR tilsiter[tiab] OR roquefort[tiab] OR edam[tiab] OR colby[tiab] OR "monterey jack"[tiab] OR provolone[tiab] OR asiago[tiab] OR romano[tiab] OR jarlsberg[tiab] OR caciocavallo[tiab] OR appenzeller[tiab] OR reblochon[tiab] OR taleggio[tiab] OR "murcia al vino"[tiab] OR gorgonzola[tiab] OR limburger[tiab] OR livarot[tiab] OR feta[tiab] OR gloucester[tiab] OR neufchatel[tiab])
#2 (Return: ‹n_2›)	(ripened[tiab] OR ripening[tiab] OR aged[tiab] OR aging[tiab] OR ageing[tiab] OR affinage[tiab] OR "flavor development"[tiab] OR "flavour development"[tiab] OR "texture development"[tiab] OR mature[tiab] OR maturity[tiab] OR maturation[tiab] OR matured[tiab] OR maturing[tiab] OR "surface-ripened"[tiab] OR proteolysis[MeSH Terms] OR proteolysis[tiab] OR lipolysis[MeSH Terms] OR lipolysis[tiab] OR "enzymatic activity"[tiab] OR "chemical composition"[tiab] OR "volatile compounds"[tiab] OR "biochemical markers"[tiab] OR "casein breakdown"[tiab])
#3 (Return: ‹n_3›)	(spectroscopy[MeSH Terms] OR spectroscopy[tiab] OR "near infrared analysis"[tiab] OR nir[tiab] OR "nir-based"[tiab] OR "infrared reflectance"[tiab] OR "fourier transform infrared"[tiab] OR ftir[tiab] OR "fourier transform near-infrared"[tiab] OR "ft-nir"[tiab] OR "visible and near infrared"[tiab] OR vnir[tiab] OR "mid-infrared"[tiab] OR mir[tiab] OR nirs[tiab] OR "short-wave near-infrared"[tiab] OR "short-wave nir"[tiab] OR "sw-nir"[tiab] OR swnir[tiab] OR "multivariate analysis"[tiab])
#4 (Return: ‹n_4›)	(industry[MeSH Terms] OR industry[tiab] OR industrial[tiab] OR "industrial-scale"[tiab] OR "scale production"[tiab] OR "production in scale"[tiab] OR "production-scale"[tiab] OR "commercial production"[tiab] OR "commercial-scale"[tiab] OR "plant-scale production"[tiab] OR "cheese plant"[tiab] OR "cheese factory"[tiab] OR manufacturing[tiab] OR "quality control"[tiab] OR "dairy plant"[tiab] OR "dairy processing facility"[tiab] OR "milk processing plant"[tiab] OR "food technology"[tiab] OR "automated analysis"[tiab] OR "real-time monitoring"[tiab] OR "process analytical technology"[tiab])
#5 (Return: ‹n_5›)	(#1) AND (#2) AND (#3) AND (#4)
	**Search refinement**: ⟨⟨Not applied⟩⟩ **Return after refinement**: ‹*N*› studies in the test carried out in ‹*date*›.

Note: MeSH is acronymous of Medical Subject Headings.

Embase™ via native interface	
**Blocks and returns**	**Search strings**
#1<br>(Return: $\langle n_1 \rangle$)	('cheese'/exp OR cheese:ti,ab,kw OR 'dairy'/exp OR dairy:ti,ab,kw OR 'mozzarella'/exp OR mozzarella:ti,ab,kw OR 'rennet'/exp OR rennet:ti,ab,kw OR camembert:ti,ab,kw OR 'cheddar'/exp OR cheddar:ti,ab,kw OR brie:ti,ab,kw OR stilton:ti,ab,kw OR gouda:ti,ab,kw OR parmesan:ti,ab,kw OR 'parmigiano-reggiano':ti,ab,kw OR parmigiano:ti,ab,kw OR emmental:ti,ab,kw OR emmentaler:ti,ab,kw OR gruyère:ti,ab,kw OR comté:ti,ab,kw OR manchego:ti,ab,kw OR pecorino:ti,ab,kw OR tilsit:ti,ab,kw OR tilsiter:ti,ab,kw OR roquefort:ti,ab,kw OR edam:ti,ab,kw OR colby:ti,ab,kw OR 'monterey jack':ti,ab,kw OR provolone:ti,ab,kw OR asiago:ti,ab,kw OR romano:ti,ab,kw OR jarlsberg:ti,ab,kw OR caciocavallo:ti,ab,kw OR appenzeller:ti,ab,kw OR reblochon:ti,ab,kw OR taleggio:ti,ab,kw OR 'murcia al vino':ti,ab,kw OR gorgonzola:ti,ab,kw OR limburger:ti,ab,kw OR livarot:ti,ab,kw OR feta:ti,ab,kw OR gloucester:ti,ab,kw OR neufchatel:ti,ab,kw)
#2<br>(Return: $\langle n_2 \rangle$)	(ripened:ti,ab,kw OR 'ripening'/exp OR ripening:ti,ab,kw OR 'aged'/exp OR aged:ti,ab,kw OR 'aging'/exp OR aging:ti,ab,kw OR ageing:ti,ab,kw OR affinage:ti,ab,kw OR 'flavor development':ti,ab,kw OR 'flavour development':ti,ab,kw OR 'texture development':ti,ab,kw OR mature:ti,ab,kw OR 'maturity'/exp OR maturity:ti,ab,kw OR 'maturation'/exp OR maturation:ti,ab,kw OR matured:ti,ab,kw OR maturing:ti,ab,kw OR 'surface-ripened':ti,ab,kw OR 'proteolysis'/exp OR proteolysis:ti,ab,kw OR 'lipolysis'/exp OR lipolysis:ti,ab,kw OR 'enzymatic activity':ti,ab,kw OR 'chemical composition':ti,ab,kw OR 'volatile compounds':ti,ab,kw OR 'biochemical markers':ti,ab,kw OR 'casein breakdown':ti,ab,kw)
#3<br>(Return: $\langle n_3 \rangle$)	('spectroscopy'/exp OR spectroscopy:ti,ab,kw OR nir:ti,ab,kw OR 'near-infrared analysis':ti,ab,kw OR 'near infrared analysis':ti,ab,kw OR 'nir-based':ti,ab,kw OR 'infrared reflectance':ti,ab,kw OR 'fourier transform infrared':ti,ab,kw OR ftir:ti,ab,kw OR 'fourier transform near-infrared':ti,ab,kw OR 'ft-nir':ti,ab,kw OR 'visible and near infrared':ti,ab,kw OR vnir:ti,ab,kw OR 'mid-infrared':ti,ab,kw OR mir:ti,ab,kw OR nirs:ti,ab,kw OR 'short-wave near-infrared':ti,ab,kw OR 'short-wave nir':ti,ab,kw OR 'sw-nir':ti,ab,kw OR swnir:ti,ab,kw OR 'multivariate analysis':ti,ab,kw)
#4<br>(Return: $\langle n_4 \rangle$)	('industry'/exp OR industry:ti,ab,kw OR industrial:ti,ab,kw OR 'industrial-scale':ti,ab,kw OR 'scale production':ti,ab,kw OR 'production in scale':ti,ab,kw OR 'production-scale':ti,ab,kw OR 'commercial production':ti,ab,kw OR 'commercial-scale':ti,ab,kw OR 'plant-scale production':ti,ab,kw OR 'cheese plant':ti,ab,kw OR 'cheese factory':ti,ab,kw OR 'manufacturing'/exp OR manufacturing:ti,ab,kw OR 'quality control'/exp OR 'quality control':ti,ab,kw OR 'dairy plant':ti,ab,kw OR 'dairy processing facility':ti,ab,kw OR 'milk processing plant':ti,ab,kw OR 'food technology'/exp OR 'food technology':ti,ab,kw OR 'automated analysis'/exp OR 'automated analysis':ti,ab,kw OR 'real time monitoring'/exp OR 'real-time monitoring':ti,ab,kw OR 'process analytical technology':ti,ab,kw)
#5<br>(Return: $\langle n_5 \rangle$)	(#1) AND (#2) AND (#3) AND (#4)
	**Search refinement**:<br>⟨⟨Not applied⟩⟩<br>**Return after refinement**:<br>$\langle N \rangle$ studies in the test carried out in ⟨*date*⟩.

Food Science and Technology Abstracts (FSTA™) via EBSCOhost interface

Blocks and returns	Search strings
#1<br>(Return: ‹n_1›)	TI (cheese OR dairy OR mozzarella OR rennet OR camembert OR cheddar OR brie OR stilton OR gouda OR parmesan OR parmigiano OR "parmigiano-reggiano" OR emmental OR emmentaler OR gruyere OR comté OR manchego OR pecorino OR tilsit OR tilsiter OR roquefort OR edam OR colby OR "monterey jack" OR provolone OR asiago OR romano OR jarlsberg OR caciocavallo OR appenzeller OR reblochon OR taleggio OR "murcia al vino" OR gorgonzola OR limburger OR livarot feta OR gloucester OR neufchatel) OR AB (cheese OR dairy OR mozzarella OR rennet OR camembert OR cheddar OR brie OR stilton OR gouda OR parmesan OR parmigiano OR "parmigiano-reggiano" OR emmental OR emmentaler OR gruyere OR comté OR manchego OR pecorino OR tilsit OR tilsiter OR roquefort OR edam OR colby OR "monterey jack" OR provolone OR asiago OR romano OR jarlsberg OR caciocavallo OR appenzeller OR reblochon OR taleggio OR "murcia al vino" OR gorgonzola OR limburger OR livarot OR feta OR gloucester OR neufchatel) OR KW (cheese OR dairy OR mozzarella OR rennet OR camembert OR cheddar OR brie OR stilton OR gouda OR parmesan OR parmigiano OR "parmigiano-reggiano" OR emmental OR emmentaler OR gruyere OR comté OR manchego OR pecorino OR tilsit OR tilsiter OR roquefort OR edam OR colby OR "monterey jack" OR provolone OR asiago OR romano OR jarlsberg OR caciocavallo OR appenzeller OR reblochon OR taleggio OR "murcia al vino" OR gorgonzola OR limburger OR livarot OR feta OR gloucester OR neufchatel)
#2<br>(Return: ‹n_2›)	TI (ripened OR ripening OR aged OR aging OR ageing OR affinage OR "flavor development" OR "flavour development" OR "texture development" OR mature OR maturity OR maturation OR matured OR maturing OR "surface-ripened" OR proteolysis OR lipolysis OR "enzymatic activity" OR "chemical composition" OR "volatile compounds" OR "biochemical markers" OR "casein breakdown") OR AB (ripened OR ripening OR aged OR aging OR ageing OR affinage OR "flavor development" OR "flavour development" OR "texture development" OR mature OR maturity OR maturation OR matured OR maturing OR "surface-ripened" OR proteolysis OR lipolysis OR "enzymatic activity" OR "chemical composition" OR "volatile compounds" OR "biochemical markers" OR "casein breakdown") OR KW (ripened OR ripening OR aged OR aging OR ageing OR affinage OR "flavor development" OR "flavour development" OR "texture development" OR mature OR maturity OR maturation OR matured OR maturing OR "surface-ripened" OR proteolysis OR lipolysis OR "enzymatic activity" OR "chemical composition" OR "volatile compounds" OR "biochemical markers" OR "casein breakdown")
#3<br>(Return: ‹n_3›)	TI (spectroscopy OR "near infrared analysis" OR nir OR "nir-based" OR "infrared reflectance" OR "fourier transform infrared" OR ftir OR "fourier transform near-infrared" OR "ft-nir" OR "visible and near infrared" OR vnir OR "mid-infrared" OR mir OR nirs OR "short-wave near-infrared" OR "short-wave nir" OR "sw-nir" OR swnir OR "multivariate analysis") OR AB (spectroscopy OR "near infrared analysis" OR nir OR "nir-based" OR "infrared reflectance" OR "fourier transform infrared" OR ftir OR "fourier transform near-infrared" OR "ft-nir" OR "visible and near infrared" OR vnir OR "mid-infrared" OR mir OR nirs OR "short-wave near-infrared" OR "short-wave nir" OR "sw-nir" OR swnir OR "multivariate analysis") OR KW (spectroscopy OR "near infrared analysis" OR nir OR "nir-based" OR "infrared reflectance" OR "fourier transform infrared" OR ftir OR "fourier transform near-infrared" OR "ft-nir" OR "visible and near infrared" OR vnir OR "mid-infrared" OR mir OR nirs OR "short-wave near-infrared" OR "short-wave nir" OR "sw-nir" OR swnir OR "multivariate analysis")
#4<br>(Return: ‹n_4›)	TI (industry OR industrial OR "industrial-scale" OR "scale production" OR "production in scale" OR "production-scale" OR "commercial production" OR "commercial-scale" OR "plant-scale production" OR "cheese plant" OR "cheese factory" OR manufacturing OR "quality control" OR "dairy plant" OR "dairy processing facility" OR "milk processing plant" OR "food technology" OR "automated analysis" OR "real-time monitoring" OR "process analytical technology") OR AB (industry OR industrial OR "industrial-scale" OR "scale production" OR "production in scale" OR "production-scale" OR "commercial production" OR "commercial-scale" OR "plant-scale production" OR "cheese plant" OR "cheese factory" OR manufacturing OR "quality control" OR "dairy plant" OR "dairy processing facility" OR "milk processing plant" OR "food technology" OR "automated analysis" OR "real-time monitoring" OR "process analytical technology") OR KW (industry OR industrial OR "industrial-scale" OR "scale production" OR "production in scale" OR "production-scale" OR "commercial production" OR "commercial-scale" OR "plant-scale production" OR "cheese plant" OR "cheese factory" OR manufacturing OR "quality control" OR "dairy plant" OR "dairy processing facility" OR "milk processing plant" OR "food technology" OR "automated analysis" OR "real-time monitoring" OR "process analytical technology")
#5<br>(Return: ‹n_5›)	(#1) AND (#2) AND (#3) AND (#4)
	**Search refinement**:<br>⟨⟨Not applied⟩⟩<br>**Return after refinement**:<br>‹*N*› studies in the test carried out in ‹*date*›.
