# Supplementary material for: Assessment of industrial cheese ripening using near infrared spectroscopy technique: A scoping review protocol
Source: PLoS One. 2025 Nov 6;20(11):e0335523. doi: 10.1371/journal.pone.0335523 (PMC12591493; doi:10.1371/journal.pone.0335523)
Supplement: S3 Table — (PDF) [file pone.0335523.s003.pdf]

**Table S3.** Data extraction summary form of the studies included in this scoping review

| (A) Citation detail | (B) Study location | (C) Study Objective | (D) Type of cheese | (E) Scale of production | (F) Stage of ripening analyzed | (G) NIR technique used | (H) Study Method (data analysis methods) | (I) Equipment and configuration description | (J) Parameters assessed during ripening | (K) Main Results | (L) Identified gaps | (M) Limitations and Gaps reported | (N) Implications and/or recommendations |
|---|---|---|---|---|---|---|---|---|---|---|---|---|---|
| Gómez & Ruiz (2020) | Spain | Evaluate inline NIR system performance in a real industrial cheese plant. | Emmental | Industrial | Continuous (real-time over 6 months) | FT-NIR (inline) | Time-series regression, moving average filters. | Foss XDS, inline, 800–2500 nm, installed on conveyor belt. | - Dry matter<br>- Salt contente<br>- Texture | Reliable real-time monitoring with minor calibration drift. | i) Lack of studies validating NIR for long-ripening cheeses.<br><br>ii) Lack of spectral standardization.<br><br>iii) Long-term stability of models under industrial noise. | • Instrument contamination and need for frequent recalibration<br><br>• High model performance but sensitive to temperature variation | a) Validating models across different production lots and integrating sensors into ripening chambers<br><br>b) Self-calibrating systems and automated cleaning protocols for sensors |

**Note:** * Hypothetical example data.
